# Can Person-Centred, Strength Based Programmes Impact on Parents’ Engagement in Education?

**DOI:** 10.1007/s41042-021-00054-y

**Published:** 2021-07-05

**Authors:** Suzanne Wilson

**Affiliations:** Westlakes Campus, University of Central Lancashire, Samuel Lindow Building, Westlakes Science and Technology Park, Moor Row, Cumbria, CA24 3JY UK

**Keywords:** Parental engagement, Educational attainment gap, Educational inequalities, Parenting programmes

## Abstract

Parental responsibility is often the focus of research and policy surrounding closing the attainment gap between low-income students and their wealthier peers. This article describes a pilot intervention programme which aimed to enable better parental support of their children with their schoolwork and educational engagement. Through interviews with the parents and facilitators involved with the pilot, this article provides an example of how strength-based interventions can promote parental engagement in education in marginalised groups, such as families considered disadvantaged. The programme consisted of six one-to-one sessions with 25 parents. Semi-structured interviews with five parents and four facilitators revealed that parents reported increased self-efficacy and confidence in supporting their children’s education. Key features of the programme contributing to sustainable changes were the programmes person-centred approach and the use of strength-based strategies. The qualitative analysis provides only short-term accounts of behavioral change, but despite these shortcomings the results provide tentative evidence for the efficacy of a brief solution focused programme in supporting low-income parents’ engagement. More research is needed using larger sample sizes with longer data collection periods.

## Introduction

Education, historically considered “the great equaliser” (Growe & Montgomery, 2003) has undergone a significant shift in many Western societies during the last 40 years. In the UK, the advent of neoliberalism saw the view of equal education for all shifting to one where individualism and marketisation prevailed, accompanied by the notion that children are parents’ responsibility rather than the state’s and thus, any failure to thrive academically or otherwise is purely the fault of parents (Crozier, 2019). This shift is most clearly illustrated through the introduction of academies. In 2020, 77% of all secondary schools and 34% pf primary schools in the UK were academy schools (Department for Education, 2021), which means although they are funded by central government, they are charitable trusts and operate independently of the government, providing more freedom over how the curriculum and how the funds are spent. There is concern that this example of neoliberal education policy may exacerbate gender, class and racial inequalities through a results-driven ethos (Kulz, 2017; Lewis & Pearce, 2020). This differs from perspectives of childhood and education promoted elsewhere such as in Scandinavian countries whose policies towards education reflect a social justice paradigm (Hicks, 2015; Tjeldvoll, 2013; see Crehan, 2017 for an international review of the policies and practices of highly performing countries).

Within this context, parents increasingly are a focus of research and policy surrounding closing the attainment gap, both in the UK and internationally (Sammons et al., 2016). Education in the UK and other countries such as US currently espouses a neoliberal context where the responsibility for a child’s academic development is placed on parents, where the “political economy of parenting” (Jensen, 2018, p. xiii) promotes constructs of ‘good’ and ‘bad parenting. Parenting interventions as a means to rescue children from ‘sub-optimal parenting’ (Gillies et al., 2017 p. 115) feature in recent political contexts, which neglect structural barriers, including political agenda impacting mostly on disadvantaged and marginalized members of society who find it difficult to thrive in a neo-liberalist environment (Goodall, 2019). Social inequalities are perpetuated by such discourses around early years parenting, pathologizing parenting preferences that do not fit within socially constructed middle-class, concerted cultivated model of parenting (Gillies et al., 2017). In an attempt to “remodel” parents (Hartas, 2014 p. 71), government policy blames parents for children’s reduced life chances, omitting the role of structural factors in shaping inequality (Hartas, 2014). This discourse continues to promote the punitive myth that “educational inequality and social immobility can be tackled through effective parenting” (Hartas, 2015 p. 32), leaving the systematic condition that create inequality and social immobility unquestioned. Working-class parents’ experiences of their involvement with their children’s education has been explored in light of class distinctions, with Crozier arguing there is a “separation between home and school”, a process she refers to as “marginalization” (Crozier, 1999 p. 320). This marginalization excludes working-class parents from obtaining the knowledge, skills and social networks to navigate the educational system. Contemporary scholars applying class analysis acknowledge that issues surrounding intersectionality, such as gender and race, can influence working-class families experience of education (Ball, 2003; Reay, 1998). Savage argued that class distinctions and inequalities are both real and powerful, fueled by a transmission of advantage powered by cultural capital (Savage, 2000). Yet within these broader structural concerns, how do we really understand the lived experiences of those subject to these forces? Beverley Skeggs, in her ethnographic study with white working-class women from North-West England, found that relentless self-doubt and self-scrutiny characterizes some working-class women’s everyday actions and decisions, who were fearful of scrutiny and the negative judgements of others (Skeggs, 1997).

One high profile intervention in the UK that aimed to support the most disadvantaged families at secondary level is the government initiative the Troubled Families Programme. The form of interventions offered to reduce truancy varied and was managed by the local authorities. The Troubled Families Programme has received much criticism, accused of generating “small, quick wins” which provide no substantial or lasting behavior change in the targeted families (Crossley, 2016 p. 142). Furthermore, others have claimed that the approach draws parallels with punitive and “antagonistic” political rhetoric such as ‘undeserving poor and the underclass’ (Crossley, 2016 p. 142). Such programmes, introduced under the auspices of support, blame parents for any social ills experienced by the families and neglect to acknowledge wider social, economic or political factors that may have contributed to the situation families are facing (Crossley & Lambert, 2017; Jensen, 2018).

This article describes a pilot intervention programme which aimed to enable better parental support of their children with their schoolwork and educational engagement. Through interviews with the parents and facilitators involved with the pilot, this article provides an example of how strength-based interventions can promote parental engagement in education in marginalised groups, such as families considered disadvantaged. The pilot project presented in this paper is derived from a larger research project that sought to better understand the perceived barriers faced by low-income families in supporting their children’s education (Wilson & McGuire, 2021; Wilson & Worsley, 2021). The research was conducted in post-industrial coastal communities in North West England, areas often referred to in policy as ‘left-behind’ (Sensier & Devine, 2017). Such communities are often characterized by high levels of deprivation, where children are vulnerable to educational isolation (Ovenden-Hope & Passy, 2019).

Parents’ accounts revealed a negative attitude towards education, characterised by a deep mistrust of theschool system and teachers. Positive attitudes to education that encourage learning within the home environment, have been demonstrated to be the most effective activities parents can do to support their children’s attainment (Hill & Tyson 2009). Parental engagement that fosters positive attitudes towards learning in the home has been shown to be most positively related to children’s achievement (Desforges & Abouchaar 2003; Sylva et al. 2003). A positive home learning environment is not simply one that provides the physical resources for learning and support with learning when needed, but also a pro-active appreciation of the value of learning which is promoted within the home. In a recent synthesis of over 800 meta-analysis of research relating to academic achievement it was found that parents can have a major effect in terms of the encouragement and expectations that they transmit to their children. Many parents, however, struggle to comprehend the language of learning and thus are disadvantaged in the methods they use to encourage their children to attain their expectations (Hattie, 2009). The impact of parental engagement in education has been shown to predict superior test scores and lower rates of dropout for youth of various racial and immigrant generation backgrounds, even in the presence of a variety of controls (Liu & White, 2017). Some international differences between parental engagement in education have been noted, for example, between Japanese and US parents, highlighting the importance to be cognizant of context when discussing parental engagement in education (Yamamoto et al., 2016). Reflecting on these results of the larger research and international literature, a brief solution focused approach (BSFA) was adopted to work with parents who reported to not engage with their children’s education.

### Theoretical Background and Application

It was important to adopt an approach that would fit the needs of the parents and of the school. The previous research with parents in this cohort revealed that the was often an issue in supporting their children’s schooling, particularly due to unsociable working hours (Wilson & McGuire, 2021; Wilson & Worsley, 2021). Brief solution focused approaches (BSFA) can be defined as “a goal-directed collaborative approach to psychotherapeutic change that is conducted through direct observation of clients’ responses to a series of precisely constructed questions” (de Shazer et al., 2007 p. 101). In a systematic review of 43 controlled outcome studies on BSFA, significant positive benefits were found for 74% of clients (Gingerich & Peterson 2013). The studies were grouped into the following categories: child academic and behavior problems, adult’s mental health, marriage and family, occupational rehabilitation, health and aging, and crime and delinquency. Out of the 14 studies in the child academic and behavior problems subgroup, 12 studies found improvements post intervention, providing tentative support for BSFA in educational settings, which support a meta-analysis showing small treatment effects of the approach (Kim, 2008). This evidence informed the rationale for using BSPA was chosen over other approaches such as mentoring, along with its direct and time limited approach, arguably making it more practically and economically appealing to academy schools who would ultimately be the market for the programme (Dameron, 2016).

The research informing the development of this intervention revealed that parents showed poor self-concept in relation to their ability to support their children with their schoolwork (Wilson & McGuire, 2021; Wilson & Worsley, 2021). Self-concept can be broadly defined as “the individual’s belief about himself or herself, including the person’s attributes and who and what the self is” (Baumeister 1999). Academic self-concept, an “individual’s perception of self with respect to their strengths, weaknesses, attitudes and values” (Phillipson & Phillipson, 2017), is central to this. There is much less evidence available on parental self-concept relating to their children’s education (Coleman & Karraker, 2000; Pelletier & Brent, 2002). In the studies that have focused on this, it has been found that parents with a stronger self-concept (self-efficacy in particular) were better educated and had higher family incomes (Boardman & Robert, 2000). Within this, perceived parental self-efficacy is defined as the “beliefs/ judgements a parent holds of their capabilities to organise and execute a set of tasks related to parenting a child” (Montigny & Lacharité, 2005 p. 394). Possible explanations include families with higher educational incomes tend to possess the necessary assets to achieve positive educational outcomes, therefore, feel more comfortable and confident in this field. The strength-based ethos within the programme was developed in response to the findings from the background research project (Wilson & McGuire, 2021; Wilson & Worsley, 2021), specifically to try to develop parents’ self-efficacy in relation to their children’s education.

A BSFA involves a strength-based philosophy, seeking to develop self-reflection of strengths and wider application of these qualities. Previous research using a strengths-based approach to parenting offer encouraging results for using self-reflective positive techniques (Jach et al., 2017; Waters & Sun, 2016). Compared with the comparison group, parents who participated in the strength-based interventions showed improved self-efficacy relating to their parenting and wellbeing. It has been argued that BFFA’s are inherently person-centred, with the focus on client self-awareness, where solutions within an individuals’ own situation can encourage clients to facilitate change (Cepeda & Davenport, 2006). A BSFA pays particular attention to the specific future goals of the individual, working with the client to map out strategies to reach these goals. A blended person-centred brief solution focused approach has been presented for school councillors, arguing that it provides a framework to focus on the student’s individual circumstance whilst limiting the time and resources required by the school (Dameron, 2016).

Furthermore, children of parents who adopt a strength-based style of parenting have demonstrated a significant effect on academic achievement which was mediated by perseverance. (Waters et al., 2019). Perseverance has been identified as a character-strengths associated with both positive classroom behavior and school achievement in both primary and secondary school students (Wagner & Ruch, 2015). Such character strengths also include construals, which have been argued to be a major source of the social class achievement gap in education (Dittmann & Stephens, 2017). It is argued that providing students from working-class backgrounds with new ways to make sense of or construe their environments can reduce the social class achievement gap. One construal-based theory this is Dweck’s Theory of Mindset (Dweck, 2006) which proposes that students, in general, have one of two distinct mindsets regarding intelligence. The first perspective is a fixed mindset, which posits that intelligence is fixed, where individuals are fearful of mistakes, so do not try. Alternatively, a growth mindset promotes the view that intelligence is flexible, where individuals take on challenges, work hard, and confront and correct mistakes. There is a wealth of supportive evidence demonstrating the efficacy of growth mindset training with children and young people (for example, Yeager & Dweck, 2012; Ahmavaara & Houston. 2007). There is less evidence detailing how different families’ mindsets may impact on parenting, but a growth mindset approach has been shown to improve children’s early gesture and vocabulary development (Rowe & Leech, 2019). This evidence informed the rationale for adopting a strength-based approach, including growth mindset, which sought to providing a meaningful and sustainable change in the attitude and behaviour of parents.

To summarise, the rationale for adopting a BSFA approach within the programme discussed in this paper was to develop a sense of self-efficacy and competence in relation to education; identify and overcome the specific challenges each parent faced in order to promote a positive attitude toward school and teachers. There is little literature documenting the use of BSFA with families, particularly within an academic context. This paper will argue that some negative attitudes and beliefs towards education and lack of self-efficacy in supporting their child’s education can be overcome using a BSFA, providing an original model of work with a strong theoretical and applied framework.

### In this Together: A Person-Centred, Strength Based Programme

In This Together aimed to develop parents’ self-efficacy in relation to their children’s education and promote positive attitudes towards education. Parents were recruited for the intervention group though liaison with a secondary academy school in a North West costal community in England. The school sent letters to families of children who were in their first year of secondary school (year 6), eligible for Pupil Premium (the additional funding that schools receive for children who receiving free school meals) and were not making expected progress against their predicted academic progress. There is debate on the efficacy of using the pupil premium as a measure for economic disadvantage (see Ilie, Sutherland & Vignoles, 2017) but for the purpose of the study discussed in this paper Pupil Premium served as a useful method for allocating participant groups and accessing school data, and is the predominant measure within educational and social science research. Parents who replied provided permission to be contacted by the principle investigator to be told more about the programme, which was followed by a phone call to discuss the project in more detail. If parents wished to participate in the programme, they then met the principle investigator in a community setting, where a participant information sheet was discussed, and a participant consent form signed. Participants were made aware of their right to withdraw from the programme and follow up interview at any time. 25 mothers participated in the pilot of In This Together (no male family members participated), including one family with twin brothers. The average age of the parent or carer was 37 years old and most described themselves as married (*n* = 12) or cohabiting (*n* = 4,). Out of the 25 participants initially recruited 23 completed the programme. Reasons given for not completing the programme were cited as being challenges around travel and lack of interest.

Facilitators received training from the programme designer detailing what was expected in each session and a one day accredited brief solution focused coaching session provided by a national organisation. The sessions were delivered within a community setting and combined worksheets with discussion. Facilitators were informed in their training that some families might have poor numeracy or literacy skills, and to use their skills to discretely offer support. Although no explicit measures of intervention fidelity were used the programme design and development underwent monthly scrutiny by academic and Inspira partners, examining progress made in forms of reports and presentations.

The table below offers an overview of the programme (Table 1):

**Table 1:** Overview of In This Together programme

WK	Title	Focus and Specific Techniques
1	MINDS	Introduction, goals for the programme agreed, growth mindset.
2	PRAISE	Positive verbal communication, effective praise, high expectations, scaling questions.
3	GROWTH	Creating a positive learning environment and learning from what we already do well, the miracle question, exceptions.
4	CHANGE	Strategies to maintain change over time, sustainable change.

In order to review progress and overcome challenges, the In This Together programme conducted follow-up sessions 8 weeks following programme completion and again at six months following programme completion. These sessions reviewed successful strategies that changed their behavior and used a solution focused approach to overcome challenges. Throughout the entire a process, a strength-based, person centred approach was the fundamental principle of the programme.

Whilst the purpose of this paper is to focus on parental experience of engaging with this programme, the impacts of the programme on student performance will be briefly outlined here. Student outcome data was obtained from the participating school which included progress, attitude, homework standard and attendance. The data from the students whose parents were participating in the programme (the intervention sample) was compared with an economically disadvantaged and non-disadvantaged sample. Results showed no decrease in progress, attitude or homework standard in the intervention sample, which were found in both control samples. Furthermore, the intervention groups’ attendance dropped at almost the same level as the non-disadvantaged sample (1.1% and 1.2% respectively), whereas the economically disadvantaged control sample dropped at a much more dramatic rate (5.4%), suggesting that the programme acted as a protective factor for attendance.

## Materials and Methods

Whilst there is tentative evidence of the positive impact this programme had on student outcomes this is not detailed here. This paper is concerned with the individual experiences of parents involved in the programme and how the programme impacted on how they saw themselves and their role in their children’s education. The following reporting has been framed within the COREQ Reporting Guidelines (Booth, et al., 2104).

### Recruitment and Sample

Purposive sampling was used to recruit participants in interviews to understand the experience of being involved in the programme. At the first programme session parents were told by the facilitators that they would be invited to be interviewed about their experiences of the programme but were under no obligation to agree. At the end of the fourth session parents were asked if they would be willing to take part in interviews and were reminded of their right to refuse and right to withdraw. If parents agreed to be interviewed, they were given a participant information form with their written invitation to participate and completed an informed consent form to complete and agreed to be contacted by the principle investigator (PI). The PI then contacted the parents to arrange a suitable time and location to hold the interviews, which were usually where the programme sessions had taken place. The same process took place with the project facilitators. Nine participants were interviewed in order to understand their experiences of being involved in the programme and their perceived impact that this had. The four facilitators who delivered the pilot were interviewed, along with five randomly selected parents who participated in the programme (Table 2).

**Table 2:** Overview of Study Sample

Parents	
Sarah (aged 37)	Sarah was a single mother of three children, aged 12, 8 and 5. Sarah was not in employment due to health reasons and had limited education. Sarah said her motivation for joining the programme was to learn how to help her children achieve their best at school, as she didn’t think she possessed the skills to do this.
Tina (aged 41)	Tina lived with her long-term partner and their children, one boy aged 16 and one girl aged 13. Both adults did not work due to mental health difficulties. Tina’s motivation for participating in the programme was to develop her ability to support her daughter’s education, particularly because she had been diagnosed with a learning difficulty and did not feel that she was being adequately supported by the school.
Lorraine (aged 31)	Lorraine was a married mother of twin boys aged 12, working part-time in a laundrette. Lorraine left school with limited educational qualifications and before embarking on the programme admitted that she struggled with confidence to support her sons’ education.
Jenny (aged 35)	Jenny was a single mother of one boy who lived with her son and her mother. Jenny left school with some educational qualifications and worked part-time as a delivery courier. Jenny described her reasons for attending the programme was to help provide her son with the support and encouragement to achieve his goal of joining the army.
Lauren (aged 42)	Lauren was married mother of three boys, aged 19, 17 and 15, and one girl aged 13. Lauren was a homemaker while her husband worked way from home. Lauren’s motivation for joining programme was to explore what she would do to support her children’s education, whilst juggling the demands of raising her children.
Facilitators	
Sharon (aged 56)	Sharon has worked for Inspira for over 20 years and was an experienced Careers Advice and Guidance Officer.
Denise (aged 49)	Denise had worked for Inspira for over 15 years and was an experienced Advice and Guidance Officer.
Jane (aged 32)	Jane had worked for Inspira for 5 years and had a youth work background.
Anita (aged 29)	Anita has worked for Inspira for 2 years and had a youth work background.

### Materials

Semi structured interview allowed for a consistent framework across all interviews whilst allowing parents and facilitators the freedom to contribute beyond the interview schedule. A similar interview schedule was used for both parents and facilitators, with the key themes being teaching methods, content, materials, effectiveness, sustainability. Both interview schedules were tested for validity with the project facilitators.

### Procedure

All interviews were carried out by the female PI who was trained in psychological research skills to Masters level and working towards a PhD. Participants had met the PI at the start of the programme and were aware of the purpose and goals of the research project. The interviews were conducted after the summer holidays, six months after completion of the four main sessions. This was to try to understand any sustainable impact of the programme on parents’ practices or their confidence. Interviews were conducted in the community and lasted approximately one hour, focusing on the experience of the programme and its perceived sustained impact. Only the participants and PI were present during the interviews, which were recorded using an audio digital recorder.

### Analytic Approach

All interviews were anonymized and transcribed, and written transcripts were analysed using the software NVivo. to The PI coded the data. Thematic analysis has been used to interpret and understand parents’ experiences of interventions to support their children (Thompson-Janes et al., 2016), where the PI identifying thematic codes which provided key generative themes emerging from the data (Braun & Clarke, 2006). Parent and facilitator data were triangulated in order to provide a coherent thematised narrative. Due to time limitations relating to this project transcripts were not returned to participants for comment and/or correction and participants were not given the opportunity to feedback on the findings.

Thematic analysis illustrates parents’ experiences of being involved in the programme, that is, how things were perceived through their life world through being data driven, identifying themes as directly through parents’ accounts, rather than a pre-identified theory which could influence interpretation of the results (Braun & Clarke, 2019). Indeed, it is not the objective facts about what specific impacts this programme produced (such as children’s academic attainment) that this paper is concerned about, it is the subjective experience of being involved in the programme and how this impacted on how parents perceive themselves. Specifically, thematic analysis was selected above other analytical frameworks based on its simplicity and theoretical flexibility (Clarke & Braun, 2014).

Pseudonyms were allocated to each participant to protect anonymity, with accounts from facilitators being indicated with an F with the pseudonym. An initial familiarising of the data revealed patterns, which was followed by the generation of initial codes to understand the experiences of engaging with the programme. These themes were then reviewed, defined and named, in order to provide a narrative framework of the parents’ experience. Key direct quotations were then extrapolated from the data and used to frame the structure of the results section.

## Results

The analysis revealed three core themes that emerged from the interviews with parents and facilitators. These were a person-centred approach, strength-based strategies and sustainable change. These will now be discussed in turn.

### A Person-Centred Approach

The attention paid to the individual families and their circumstances was felt by both parents and facilitators to be of great value. The importance of facilitator skill in building relationships and facilitating discussion can help parents reflect on their current practice, as was felt my Anita (F):

I think the more you talk with them the more they realise what they do. Sometimes, at first, there can be this kind of blank look and they’re thinking ‘I don’t think I do anything.’ Then as you talk with them, they’re like, ‘I do this and I do that.’

Here Anita illustrates how discussion can support self-reflection and confidence. There were instances when parents discovered their own solutions to their perceived problems after just one session, for example, in contacting the school to resolve an issue or devising a routine within the home. This experience is summarised in the account below:

She came after that first session with an idea of what she wanted to discuss and what she wanted to do. That first meeting that we had with her gave her confidence and she blossomed, knowing what she wanted and how she was going to do with it (Denise F)

Again, this passage provides an example of parents being able to come to their own solutions through a BSF mentoring relationship,, which resonates with previous research around BSF techniques (Jach et al., 2017; Waters & Sun, 2016) and adds to the literature arguing for its use within an educational context (Dameron, 2016).

The home-based activities to try at home were treated in a variety of ways. On the whole, “parents really welcomed to not only do stuff at home, but included the kids”, and it was felt that was:

because it was new to them, new ideas, fresh ideas, that they were willing to give them a try. Which is why they came back the next session and said, ‘Look this is what we did!’ And they were quite chuffed, quite proud at what they’d done(Jane F).

These provide examples of parents observing progress over time and effective facilitator feedback, all of which are part of the brief solution focused technique. This promoted confidence, which is central, “because parents have so little self-confidence or self-regard, and they don’t particularly think they are doing anything effective to support their children’s education” (Jane F). This strategy is illustrated when Sharon summarised:

At the end of the fourth session, he still hadn’t got into a routine, but I’ve met with her twice since then and she doesn’t need to nag anymore. She’s given him that little bit more responsibility and accountability (Sharon F).

It was felt that mentoring relationship between parent and facilitator was central to the success of the project, as Denise reflected the “rapport you can build with people makes a massive difference and it was felt that “working more intensively one to one, essentially you’re going to get a bigger impact because you can give them your attention” (Denise F). Furthermore, it was felt that this method of working “gives more ownership” as parents are:

made a bit more accountable because it’s an intense mentoring relationship...You build up such a rapport with the parent, they have complete trust in you. I think that’s part of it. Someone believes in them. You could be that one person who believes in that parent” (Jane F).

A number of parents confirmed the power of the trust described by Jane, for example Lorraine said it was “I felt I could trust her, the meetings were all about me and how I could do things differently for my son” and Sarah said how “they seemed to really care about me and my son, I could tell that from the first meeting, it helped me to feel more relaxed and able to talk about things”. The specific skills valued in the mentoring relationship were active listening with Jenny stating that she found it very helpful for “someone to be saying things back that you’re already doing, it made realise what I was doing”. By the end of the programme most parents wanted to bring their children to the sessions in order to meet the facilitator, which is testament to the trust and respect parents placed in the facilitators and shows the potential power of the mentoring relationship, even over a short period of time. All parents interviewed valued the person-centred approach, described by Jenny:

it was a case of me listening to someone else talking to me about different ways of targeting whatever you’re going to do. And listening to it from another point of view and thinking, ‘Oh well, Ok I can try that.’

Indeed, Tina said:

it was fun, I can talk to Anita, you can have a laugh with her. I think that says it all really.

The importance of informality in the mentoring relationship is particularly highlighted in the above passage, and how this can promote rapport and engagement.

### Strength- Based Strategies

The use of strength-based strategies was at the centre of the programme and included positive praise, growth mindset and exceptions. Positive Praise, that is, offering praise focused on the effort not just the outcome, was reported by some parents and facilitators has been a useful method of working with their children. Lauren summarised how she now praises her son:

I said to him, ‘You’ve done really, really well. You’ve only been there since September last year and you’ve done remarkably well.’ I said, ‘Now it’s onwards and upwards, don’t go backwards with them and just keep going on as you can.’

The success of this technique was recounted by Sharon (F), “it was like a little light bulb went off and they were like, ‘Yeah, I don’t do it, but I can do it because it’s not difficult. It’s just something that I need to expand on what I already do.”

Parents expressed that they found the growth mindset techniques helpful to support their children with their school work, for example Jenny described how, “[her son] will say his homework is rubbish and I say well how you can make it better, so it’s worked for us.” It was reflected that parents both use this technique with their children, and also on themselves

he’s said a few times, ‘Oh I can’t do that.’ And I say to him, ‘Well you never know unless you start to try. And if I can help you I will.’ So, I’ve used it like that, and I’ve said to him there’s no such word as can’t...I say that to myself as well.

The above passage summarises the simple technique, that doesn’t require academic ability. Lauren described the change in how she supports her children with their homework through using growth mindset:

I used to respond by saying, ‘I don’t know.’ Now I’m like, ‘Tell me what you do know.’ Then we go in the internet and see what we can find. When I’m doing it, we both look, and I might give her a bit help. Then she has to work out the rest.

Furthermore, Lauren highlights how she now feels able to be proactive in supporting her children. Tina reflected that she continues to use growth mindset with her children, but it can be challenging at times:

It can be hard constantly making things seem better, because she will whinge and moan and get moody about things...I try and continue to try. And I will always try, and I hope that one day she will come out of something and think that is exactly what I would have done.

Growth mindset was also a popular method with facilitators and was applied both to encourage parents and students to change their behavior. Examples of parents reporting positive changes include Jenny son’s quality of English homework improving as she was “always using growth mindset” and Sarah despite her initial reluctance, preserved and found “it really works.” Sharon reflected through covering growth mindset in the sessions, parents:

realise that, ‘actually I can do better than that. I can push my child more, I can be more positive, I can push them in the right direction and give them that little bit more structure.’ I think growth mindset really works.

This account is littered with ‘I can’ statements, which typifies the programmes focus on highlighting the important role parents have in their children’s education. These accounts build on existing evidence arguing the efficacy of parental use of growth mindset techniques in education (Yeager & Dweck, 2012; Ahmavaara & Houston. 2007).

Exceptions involves highlighting what parents are already doing to support their children and applying these successful techniques to other situations. This technique was reported to be used in all sessions by facilitators, which contributed to improving parents’ confidence:

I’m not sure she was confident initially that she could make a difference. That she could help him overcome some of the things he was struggling with. I reiterated all the way through that she was making a difference and there were things she was already doing (Denise F)

The above passage illustrates the power of the mentoring relationship, in helping parents be self-reflective of their current actions, and how to adapt these to other situations. These accounts resonate with previous findings that that BSF mentoring can encourage self0reflective behaviours in identifying areas in their lives they wanted to make improvement and in developing strategies to achieve these goals (Cepeda & Davenport, 2006).

In her first session Lorraine provided a vivid example how parents may be using effective and supportive engagement strategies when supporting their children’s education without realising it. When struggling with homework, she described how she would “just sit with [her son] and look it up on Google”. When describing this Lorraine clearly felt uncomfortable, sinking into her chair. The facilitator was able to provide reassurance that this was a valuable strategy to adopt because she is spending time with her son and they were learning together.

### Sustainable Change

Both parents and facilitators were specifically asked about any changes they had made or observed. Although facilitators could not comment on longer term impacts beyond that of the follow up sessions, they were able to share what they felt were the key ingredients to make positive, sustainable changes.

Helping with homework, which was the central concern regarding their children’s education, was noted to be something parents’ benefited from, was summarised by Tina, “it’s definitely had a positive outcome, like I said I used to say, ‘I didn’t know, ask someone else’. But I think ‘we can give it a good go.’” This was supported by Sarah:

I‘ve put quite a few little bits into place. Doing her Maths homework the other day she asked ‘Mum, did you do Algebra?’ I said, ‘only a little bit’. Then it was a case of ‘what does that mean?’, and I said, ‘well I think it might mean -2, or maybe not’. So yes, I do help quite a lot now when it comes to homework.

The above comments illustrate the degree of self-efficacy parents perceived around supporting their children with their homework, which was an issue identified by all parents as a challenge in the first session. The embedded nature of the perception of lack of self-efficacy was highlighted by Lauren who expressed that she was “not very good really” in supporting her children with their school work, but went on to describe an instance when her daughter was punished at school for cheating on her reading tests. On finding this Lauren contacted the school, asking for a list of approved books, and went shopping in charity shops for books for her daughter. She also talked to her daughter about why she wasn’t reading, which was because she struggled with reading and got confused. In response, Lauren shared with her daughter that she also struggled with reading, and worked out the following method to ensure that her daughter was doing the required work:

She had this book and she started reading it. Then she was confused. I told her to tell me every chapter what was happening. Then I was telling her ‘now you have to go to bed and read because I want to know what’s happening!’ Then she got to a bit and said, ‘Mam I’m well confused’ I said, ‘well let’s just recap and take it from there’.

Lauren then used this learning to continue to support her daughter with a reading scheme over the school holidays, again, looking for second books and talking through the books with her daughter. It must be noted that this actual technique of working with her daughter wasn’t included in the content of any session, and that this was a result of Lauren taking Lauren realising the role she had in her children’s education and using her initiative to her own skill set to support her daughter. She goes on to reflect:

Homework is a big thing I’ve never been a school person; homework had never been at the top of my list. As long as they’re happy I don’t care what they come out of school with. That’s not really the right attitude. You have to have a little bit of structure to make sure the homework is done.

The above case demonstrates how working with parents to promote confidence can positively impact on their self-efficacy relating to their children’s education, and their subsequent practice. This is particularly important when considered in the context that better educated parents tend to have a stronger education self-concept (Boardman & Robert, 2000), showing the value of interventions that promote self-efficacy in parents who are less well educated. Furthermore, these stories provided by parents, where they are challenged themselves, despite lacking confidence, shows the value of perseverance, complimenting previous research on this character trait (Wagner & Ruch, 2015; Waters et al., 2019).

Facilitators expressed they felt that parents’ confidence in supporting their children in their education had been enhanced as a result of the programme, mainly from providing parents with:

the chance to see different techniques and they’ve had the chance to try them out” (Denise F).

It was felt that this confidence was gained from understanding that academic support doesn’t necessarily come from academic content:

They feel like they’ve got the knowledge there, maybe they don’t necessarily know the answer to the homework question, but they feel like they can help in some way in order for the child to keep on progressing and doing well (Jane F)

It was however felt that not all participants were able to fully self-reflect on their behavior change positively impacting on their children, as summarised Sharon (F):

she probably doesn’t recognise it herself, and she does want something better for her daughter...I think she will be gently pushing that over the next couple of years.

It was also felt the that project encouraged parents to promote aspirations in participants’ children, in:

getting them to do things that are about the future, and how they’re going to get there, both with the parent and the idea that the child’s got” (Sharon F).

This reflection highlights the role in not only identifying a future career aspiration, but also in acknowledging strategies to support children get there.

## Discussion

In This Together aimed to develop parents’ self-efficacy in relation to their children’s education and promote positive attitudes towards education and there is evidence supporting that the BFSA achieved this goal. Follow up interviews provided tangible examples of parents using the tools discussed in the sessions, seeing positive results and acknowledging they can make a difference. Results presented in this paper suggest that a strength-based approach, focused on parents’ existing assets can promote sustainable change in terms of parents confidence and involvement in their children’s education. Interviews identified three key factors that contributed to the reported impact of the intervention. These were strong, trusting mentoring relationships, that used strength-based techniques, that in turn promoted self efficacy in education. These will now be discussed.

The use of strength-based strategies provided opportunities for parents to challenge the negative self-beliefs they held about their abilities to support their children’s education. In particular, growth mindset was reported to have been the most utilised and sustainable method in ensuring behavior change in relation to educational engagement. These results support the efficacy of growth mindset training with children and young people (for example, Ahmavaara & Houston 2007; Yeager & Dweck, 2012), as parents demonstrated their use of techniques with their children to encourage positive outcomes. Furthermore, this study provides an original application of the approach by supporting parents to use a growth mindset in their parenting and also promote these values in their children, along with advocating the use of a strengths-based approach to positively engaging disenfranchised parents in their children’s’ education (Jach et al., 2017).

Accounts from participants showed the value that was placed in promoting self-efficacy in supporting their children’s education. This is within a neoliberal context where the dominant discourse argues that those from more disadvantaged backgrounds inherently lack the ability to support their children’s education. Inequalities are further exacerbated in the current neoliberal climate through a meritocratic ideology, the belief that success is an indicator of personal deservingness, rewarding individual ability and efforts (Jost et al., 2003; Wiederkehr et al., 2015). This construct has been argued to be internalised, subsequently impacting on engagement in education (Wilson & McGuire, 2021). Findings from the pilot intervention provides encouraging evidence that a strength-based approach can be used to mitigate the pervasive impact of perceived stigma in education. The insights gained through this pilot can be used to support contemporary arguments for adopting a capacities approach to promote equality in education such as those presented by del Moral-Espín and Domínguez-Serrano (2020) who uses the approach specifically in relation to wellbeing.

Research findings from the works informing this programme revealed a deep distrust and perceived disconnect from teachers, who were seen as being judgemental towards families (Wilson & McGuire, 2021; Wilson & Worsley, 2021). These perceptions were acknowledged in the development of the programme and the facilitator training, with an explicit emphasis on building trust and creating a supportive, non-threatening environment for parents. What stands out particularly in these results is how the ethos of the BSFA, not just techniques, had a major impact on the engagement in the project. Rapport, facilitator feedback and clearly formed outcomes were identified as being particularly effective in building confidence, promoting self-efficacy and behavior change in parents. Furthermore, the flexibility demonstrated by facilitators in their practice was felt to benefit engagement. This differs from other successful studies using BSFA in educational (Cepukiene & Pakrosnis, 2011; Daki & Savage, 2010; Franklin et al., 2008; Froeschle et al., 2007; Newsome, 2004; Springer et al., 2000) or family contexts (Chung & Yang, 2004; Eakes et al., 1997; Kenney, 2010; Huang 2001; Naude, 1999; Zimmerman et al., 1996), which were delivered through groups or family settings. The current study highlights the powerful impact that targeted one-to-one interventions can have in parental engagement in education, and more work is needed to identify key factors behind the intervention’s success.

### Implications

These results add to a bank of interventions to which pupil premium funds can be spent to narrow the educational gap and are suggestive of parents’ motivation to support children, but a fear and lack of knowledge of how to do so. This can be used to inform schools in successfully engaging parents. For example, the results suggest that practices that highlight and celebrate the achievements of parents, along with those of students, could promote parental engagement in education. Given the experiences of parents who participated in this pilot, it could be suggested that a whole school policy that advocates a strength-based approach to all stakeholders, including parents, could help to promote engagement in education through removing some of the perceived barriers faced by families, shaped from previous negative experience of education. In a period adapting to the Covid-19 outbreak, which has seen a dramatic shift in parental responsibility for education, strategies that remove barriers between home and school are more important than ever.

Despite these encouraging findings, it is argued that strength based, person centred approaches alone are insufficient to promote confidence and self-efficacy in parents who have faced significant challenges in the past in engaging in education, either as children themselves or as parents. The programme was piloted in the UK within a policy context that promotes parental intervention as a means of closing the attainment gap (Goodall, 2019). Recent research, however, has argued that parental engagement strategies tend to deflect attention away from the structural barriers affecting educational achievement and consequently reinforce deficit construction of certain groups of parents, such as low-income families (Fretwell, 2020; Hartas, 2014, 2015). Individualist policies with the focus on changing parental behavior neglects to acknowledge the structural barriers faced by low-income parents (Crozier et al., 2011; Goodall, 2019). The Covid-19 outbreak has intensified the role of parents in education, with children across the globe were being schooled at home which has potentially exacerbated the barriers experienced by low-income parents. With this in mind, a critical reflection on potentially punitive policies and practices that could further isolate and disengage families in education is more relevant than ever.

These results add to the building evidence that policy should prioritize reducing socio-economic differences in children’s academic performance and well-being, which can be done through building parents’ capabilities through improving access to education, secure employment and public services (Hartas, 2014). This paper proposes that the In This Together programme fits within this capacity building framework, whereby disenfranchised working-class parents may feel more confident and self-efficacious in relation to their children’s education, becoming active participants in their children’s education, thereby attempting to redress some of the power inequalities that exist.

### Limitations and Future Research

The sample size of the analysis used in this paper is very small and thus the findings cannot be generalised or used to provide definitive supportive evidence for the efficacy of the programme or the impact it had on parents. Furthermore, the interviews took place with parents and facilitators just six months following the completion of the main four programme sessions. This neglects the longer-term impact of the programme which is essential to measure and understand any programmes that attempt to promote behavior change. More research is needed to further understand the experiences of low-income parents, particularly those from ‘left behind’ communities such as post-industrial British coast towns (Sensier & Devine, 2017). No fathers or male caregivers participated in the programme and thus were not included in this analysis. It is important to better understand the reasons why no males participated to better inform programme to involve male caregivers. This would contribute to the limited literature surrounding low-income fathers and male caregivers’ attitudes and practices relating to their children’s education (David, et al. 2003). Likewise, further research with different groups and factions of the population would help to uncover the role intersectionality plays in parental engagement. The timing of the interviews did not allow for sufficient opportunity to develop sustainable long term change. A further interview, after 8 or12 months would have provided more information about any changed implemented both within term time and school holidays.

## Conclusions

Many traditional ‘parenting programmes’ are based on the neoliberal assumption that low-income parents lack the necessarily skills and capabilities to support their children’s education. In This Together provides encouraging evidence demonstrating the efficacy of using a BSFA to promote parental self-efficacy in supporting children’s education. The mentoring relationships helped to build trust and the strength-based techniques cultivated parental self-efficacy. The strategies parents adopted to support their children’s education serve to illustrate the somewhat hidden skills and assets that parents possess. It highlights the important role that context plays in the use of the skills that were always present but not applied in an educational context. A programme that acknowledges and celebrates the existing assets parents possess, along with sharing new strategies, can help to develop relationships and trust between parents and teachers, something of which benefits the child, the parent and teachers alike. Whilst the findings from this pilot are clearly of value in providing an example of a capacity-building embedded parenting intervention, educational policy and practice must also undergo some critical review and revision to promote inclusively to all families.
